# Enhancing the Stability of LiNi_0.5_Mn_1.5_O_4_ by Coating with LiNbO_3_ Solid-State Electrolyte: Novel Chemically Activated Coating Process versus Sol-Gel Method

**DOI:** 10.3390/nano11020548

**Published:** 2021-02-22

**Authors:** Valeriu Mereacre, Pirmin Stüble, Ahmad Ghamlouche, Joachim R. Binder

**Affiliations:** 1 Institute for Applied Materials, Energy Storage Systems, Karlsruhe Institute of Technology, Hermann-von-Helmholtz-Platz 1, D-76344 Eggenstein-Leopoldshafen, Germany; pirmin.stueble@kit.edu (P.S.); ahmad.ghamlouche@kit.edu (A.G.); joachim.binder@kit.edu (J.R.B.); 2 Helmholtz Institute Ulm, D-89081 Ulm, Germany

**Keywords:** LiNbO_3_, X-ray diffraction, lithium-ion batteries, cathode materials, electrochemical properties

## Abstract

LiNbO_3_-coated LiNi_0.5_Mn_1.5_O_4_ spinel was fabricated by two methods: using hydrogen-peroxide as activating agent and sol-gel method. The structure of the obtained cathode materials was investigated using a scanning electron microscope (SEM), X-ray diffraction (XRD), X-ray photoelectron spectroscopy (XPS), and the electrochemical properties of the prepared cathodes were probed by charge-discharge studies. The morphology of the coating material on the surface and the degree of coverage of the coated particles were investigated by SEM, which showed that the surface of LiNi_0.5_Mn_1.5_O_4_ particles is uniformly encapsulated by lithium innovate coating. The influence of the LiNbO_3_ coating layer on the spinel’s properties was explored, including its effect on the crystal structure and electrochemical performance. XRD studies of the obtained coated active materials revealed very small expansion or contraction of the unit cell. From the capacity retention tests a significant improvement of the electrochemical properties resulted when a novel chemically activated coating process was used. Poorer results, however, were obtained using the sol-gel method. The results also revealed that the coated materials by the new method exhibit enhanced reversibility and stability compared to the pristine and reference ones. It was shown that the morphology of the coating material and possible improvement of communication between the substrates play an important role.

## 1. Introduction

One of the most promising candidates for cathode material in high-voltage rechargeable lithium-ion batteries is LiNi_0.5_Mn_1.5_O_4_ spinel (LNMO) [1,2,3,4]. Its increased working voltage has made this material very important for the production of cathode active materials with enhanced energy density [5]. However, the practical use of this spinel has so far been hindered by some drawbacks [6]. The low interfacial stability between the cathode and the electrolyte slows down the widespread practical use of these materials [7,8]. In the crystals of this kind of spinel there is usually a small amount of Mn^3+^ ions. Thus, the weakness is owing to the presence of Mn^3+^ in the structure of the LNMO and is considered to be one of the main causes for the short cycling and storage life of a cell. Because of the disproportionation reaction of Mn^3+^, the Mn^2+^ ions are obtained. These ions have the tendency to dissolve into the electrolyte, in this way accelerating the capacity fade of the cell [9,10,11]. In order to overwhelm the dissolution of manganese ions [12] and increase the electrochemical performance of such cathode materials, their surface is covered with a coating layer [13,14,15,16], which acts as a passivation layer, preventing the active material from direct contact with electrolyte.

In this work, LiNbO_3_ was chosen as coating material, because it has a very good structural stability and a high room-temperature lithium ionic (up to 10^−2^ mS/cm) and low electronic (up to 10^−8^ mS/cm) conductivity [17]. In addition, LiNbO_3_ displays good conductivity not only in the crystalline, but also in amorphous form. This should allow coating cathode materials with amorphous constituents, which can improve not only the stability, but also the synthesis of such materials that require “softer” conditions. However, the protection properties and good conductivity of the coating material are not enough properties for a good coating layer. In addition to these properties, a good contact between active material and the coating layer should be achieved.

The coating of cathode materials with Li/Nb-containing compounds is well documented in the literature [18,19,20]. The key methods for obtaining such coatings are solid state reactions [18] and sol-gel methods [19]. However, a homogeneous and complete coating layer on the host materials is not always achieved when using these techniques. Solid-state reactions are widely used processes, but they result in particle morphologies which are difficult to control. Because the coated material is produced from a chemically homogeneous precursor, the sol-gel methods show some specific benefits. However, the obtained materials often have areas that are better and others that are less protected from side reactions with the electrolyte.

In this study, LiNbO_3_-coated LNMO was synthesized by a simple, widely used sol-gel method and by a novel, fast and low-cost synthetic approach. The effect of the LiNbO_3_ coating layer on the LNMO spinel cathode was studied, comprising the impact of the coating material on the crystal structure and electrochemical performance. It seems that when using the new synthetic method, a LiNbO_3_ coating layer with improved effectivity against Mn dissolution was obtained. Scanning electron microscope (SEM), X-ray photoelectron spectroscopy (XPS) and X-ray diffraction (XRD), combined with electrochemical property measurements were carried out on both the pristine LMNO sample and the LiNbO_3_-coated samples.

It is worth mentioning that the new coating method might also be useful for the investigations in the field of ceramic solid electrolytes for the rechargeable lithium batteries. The major problem claimed in this field is poor contact between solid electrolyte and active material resulting in increase of the charge transfer resistance and cell impedance. Using our method, it is possible to circumvent this problem: surface activation and simultaneous coating results in a much better contact and communication between the coated layer and active material. Obviously, there are other candidates/ceramics with much higher ionic conductivities than in LiNbO_3_, which can be used as solid-state electrolytes. However, the practical material for solid-state battery applications may not be that with the highest conductivity, but the one which can be prepared inexpensively in a homogeneous and thin-film form directly onto the cathode in this way, enhancing the interfacial contact.

## 2. Materials and Methods

### 2.1. Fabrication of LNMO Precursor

The precursor for LNMO was prepared by a co-precipitation method reported elsewhere [21]. In a typical synthesis route, 0.185 mol of analytical reagent grade LiCH_3_COO⋅2H_2_O, 0.090 mol Ni(CH_3_COO)_2_⋅4H_2_O, and 0.270 mol Mn(CH_3_COO)_2_⋅4H_2_O (all supplied by Alpha Aesar, Kandel, Germany) were dissolved in 300 mL of water. The solution was transferred into a 1 L continuously stirred tank reactor [22]. Separately, 0.451 mol of H_2_C_2_O_4_⋅2H_2_O were dissolved in 450 mL of water. Under temperature of 50 °C and constant stirring (400 rpm) of the Li-Ni-Mn solution, 30 g of solid PEG6000 was added. After 10 min, when PEG6000 dissolved completely, the oxalic acid aqueous solution was pumped (10 mL/min) into the continuously stirred tank reactor. After the oxalic acid solution was completely pumped, the reaction mixture was stirred for additional 20 min. Then, the obtained suspension was dried at 90 °C to obtain a green viscous precursor (~85 g). The resultant precursor was calcined in air (air flow 5L/min) at 450 °C for 4 h and then at 900 °C for 24 h, and cooled down to room temperature with the heating and cooling rate of 4 °C/min to obtain the LiMn_1.5_Ni_0.5_O_4_, which is labelled as LNMO. Brunauer-Emmett-Teller (BET) surface area was 0.3 m^2^g^−1^. The reference compound, LNMO-800 was obtained by calcining pristine LNMO in air at 800 °C using the following program: 250 °C for 2 h and then heated at 800 °C for 10 h, and cooled down to room temperature with the heating and cooling rate of 4 °C/min.

### 2.2. Preparation of Li/Nb Coating Solution

Reagent-grade lithium ethoxide (LiOC_2_H_5_) and niobium ethoxide (Nb(OC_2_H_5_)_5_) (both supplied by Aldrich, Steinheim, Germany) were used as lithium and niobium sources. Two solutions were prepared: 0.26 g (0.5 mmol) LiOC_2_H_5_ were dissolved in 20 mL water free (absolute) ethanol. Separately, 0.15 g (0.47 mmol) Nb(OC_2_H_5_)_5_ was dissolved in 10 mL water free (absolute) ethanol. Under magnetic stirring of the first solution, the second one was added. To the obtained mixture, 30 mL of H_2_O_2_ (30%) was added and the resulting mixture was stirred for additional 10 min. 

### 2.3. Material Coating: Sol-Gel mEthod

In a round bottom flask (100 mL) with 0.5 g LNMO, under stirring, 7 mL water free (absolute) ethanol was added. After ~5 min, 2.15 mL Li/Nb coating solution was added. After an additional 2 h of stirring, the reaction mixture was transferred in a glass (V = 100 mL) and dried during one hour at 110 °C. The obtained dry powder was divided in six portions and calcined in air at T = 300, 400, 500, 600, 700 and 800 °C using the following program: 250 °C for 2 h and then heated at particular T °C for 10 h, and cooled down to room temperature with the heating and cooling rate of 4 °C/min. The quantity of the LiNbO_3_ expected to be coated on the surface of LNMO particles was around 0.5 wt.%. The obtained samples were labelled as LNMO-LNbO-300, LNMO-LNbO-400, LNMO-LNbO-500, LNMO-LNbO-600, LNMO-LNbO-700 and LNMO-LNbO-800, respectively.

### 2.4. Material Coating: New Chemically Activated Coating Method

In a round bottom flask (100 mL) with 0.3 g LNMO, under stirring, 10 mL H_2_O_2_ (30%) was added. After ~20 s, 1.3 mL Li/Nb solution was added. During the first 20 min the reaction was relatively silent, but after this time, the reaction started to be very violent with the forming of a white fog. The violent reaction took place in less than 2 min. After an additional 5 min of stirring, the reaction mixture was decanted and the obtained powder washed with 25 mL water and dried for one hour at 100 °C. The obtained dry powder was calcined in air at T = 800 °C using the following program: 250 °C for 2 h and then heated at 800 °C for 10 h, and cooled down to room temperature with the heating and cooling rate of 4 °C/min. The quantity of the LiNbO_3_ expected to be coated on the surface of LNMO particles was around 0.5 wt.%. The obtained sample was labelled as LNMO-nLNbO-800. A similar reaction was made with 6.5 mL Li/Nb solution, resulting in LNMO particles coated with approximately 2.5 wt.% LiNbO_3_. The obtained sample was labelled as LNMO-nLNbO-800(2.5%).

### 2.5. Coating Preparation

A total of 50 mL of the Li/Nb coating solution was used to prepare pure LiNbO_3_ in crystalline form. Under stirring, the solution was heated in a glass on a hot plate at about 100 °C until a dry powder was obtained. Crystalline LiNbO_3_ powder was obtained by calcination at 800 °C in air and labelled as LNbO-800. The calcination program was the same as for the coated LNMO-LNbO samples.

### 2.6. Powder X-ray Diffraction and Scanning Electron Microscope

Powder X-ray diffraction (XRD) for the samples LNbO-800, LNMO, LNMO-800, LNMO-LNbO-800, LNMO-nLNbO-800 and LNMO-nLNbO-800(2.5%) was carried out using a STOE diffractometer (STADI, Darmstadt, Germany) equipped with a Cu X-ray tube and a diffracted beam monochromator. The scattering angle (2θ) range was 10–80° at 0.03° intervals with a dwell time of 10 s per point. A scanning electron microscope (SEM, Zeiss Supra 55, Oberkochen, Germany) was used to elucidate the morphology of precursor, coating material, and coated samples. Before measurement the samples were prepared by mounting the powder on adhesive carbon tape.

### 2.7. X-ray Photoelectron Spectroscopy

XPS spectra were acquired using a Thermo Scientific K-alpha spectrometer (Thermo Fisher Scientific GmbH, Dreieich, Germany. The samples were analyzed using a micro focused, monochromatic Al K_α_ X-ray source (1486.6 eV, 400 μm spot size). The spectra were recorded with a concentric hemispherical analyzer at a pass energy of 50 eV.

### 2.8. Electrochemical Measurements

Electrochemical measurements were carried out via galvanostatic charge/discharge cycling using 2032 coin cells with lithium metal as the anode on a BT2000 battery cycler (Arbin Instruments, College Station, TX, USA). During electrode preparation, the slurry was a mixture of 80 wt.% active material, 10 wt.% Super-S carbon black (Timcal, MTI Corporation, Richmond, CA, USA) and 10 wt.% PVDF (Polyvinylidene fluoride) with NMP (1-Methyl-2-pyrrolidinone) as solvent. Electrodes were prepared by coating the slurry on an Al foil with a 200 μm notch bar spreader and dried in air at 80 °C for 20 min, then at 100 °C for 24 h in vacuum. Usual cathode loadings were in the range of 4.0–5.0 mg⋅cm^−2^; an electrode diameter of 12 mm was used. Before use, the cathode disks were pressed in a hydraulic press to 6 kN and dried additionally for 60 min in a vacuum oven at 110 °C. The electrolyte was 1.0 M LiPF_6_ solution in 1:1 *v*/*v* ethylene carbonate:diethyl carbonate (EC:DEC). A lithium metal foil (diameter 12 mm) was used as anode. One layer of Celgard 2325 (Celgard, Sélestat, France) on the lithium and one layer on the positive electrode were used as separators, with one GFC microfiber separator in the middle. Cells were assembled in a dry Ar-filled glove box. Cycling was performed at a temperature of 23 °C. A voltage window of 3.5–5.0 V versus Li^+^/Li was applied. Electrochemical impedance spectra (EIS) were measured with an AC voltage of 5 mV amplitude in the frequency range of 500 kHz to 10 mHz using a Biologic (Seyssinet-Pariset, France) potentiostat instrument.

## 3. Results and Discussion

It is well known that the sol-gel technique is an important preparation method for inorganic materials and is frequently used for preparation of different oxide powder products [23,24,25,26]. Such materials usually have high purity and are possible to prepare at temperatures far below their melting point. As result, one can produce a large number of materials of different shapes and morphologies, which are difficult to obtain by conventional methods, due to their high melting temperatures. This method is also commonly used for coating of battery materials [27,28].

In this work, the LiNbO_3_ powder was obtained using a sol-gel method and the identical temperature handling as that for coated LNMO: under stirring the solution containing Li and Nb ions was heated at about 100 °C until a gel and later a dry powder was obtained and calcined at 800 °C. In contrast to pure LiNbO_3_, the LiNi_0.5_Mn_1.5_O_4_ coated with LiNbO_3_ oxide was synthesized by two methods: sol-gel and a new method using hydrogen peroxide as activating agent.

The X-ray studies of the coating material obtained at 800 °C (Figure S1) indicate that the material is a crystalline oxide. Relatively narrow reflections are observed and can be indexed to LiNbO_3_ (~83%) and Li_3_NbO_4_ (~17%) phases. Several additional weak reflections indicate the presence of a small quantity of a substance with an unknown structure. Hence, the most plausible component of the coating layer on the surface of LNMO particles is anticipated to be LiNbO_3_. 

**Figure 1:**
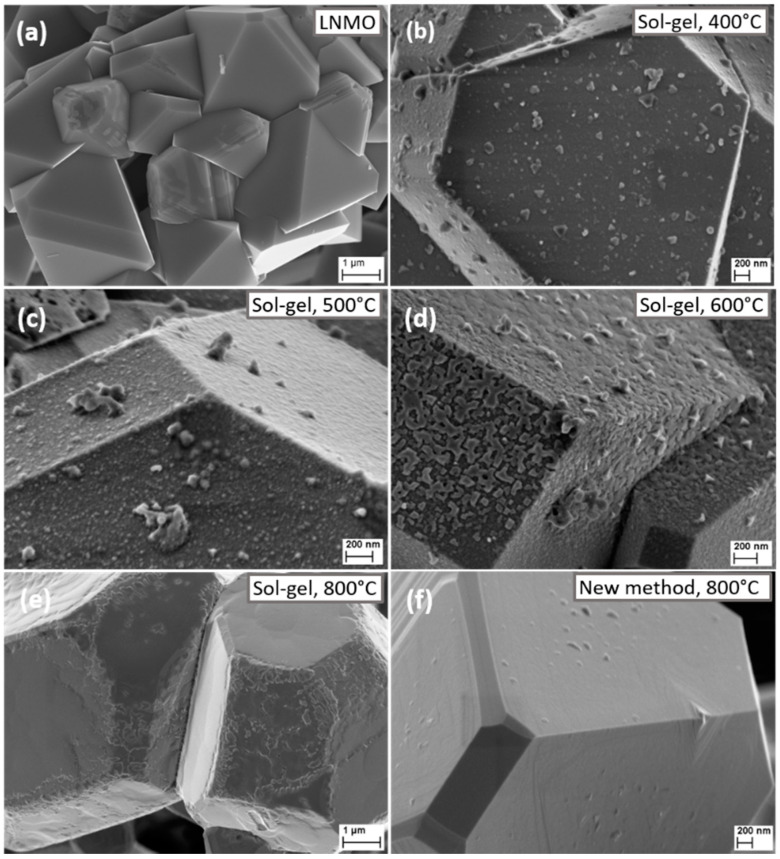
Scanning electron microscope (SEM) diagrams of samples (**a**) bare LMNO, (**b**) LMNO-LNbO-400, (**c**) LMNO-LNbO-500, (**d**) LMNO-LNbO-600, (**e**) LMNO-LNbO-800 and (**f**) LMNO-nLNbO-800.

**Figure 2:**
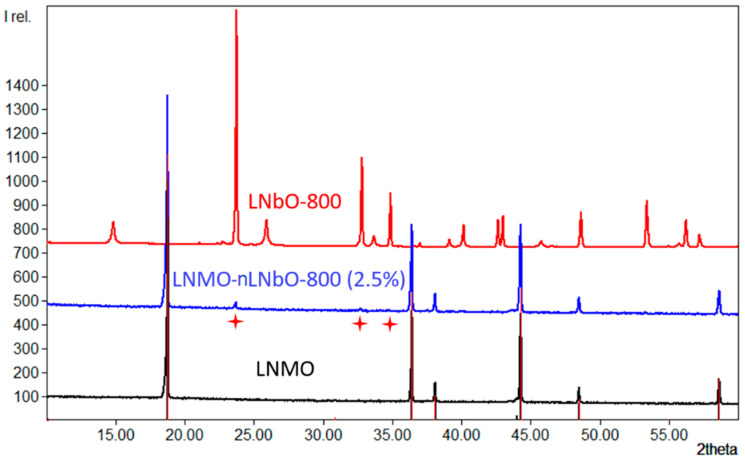
X-ray diffraction (XRD) patterns of uncoated LiMn_1.5_Ni_0.5_O_4_ (LNMO), LNMO coated with 2.5 wt% LiNbO_3_ and calcined at 800 °C (LNMO-nLNbO-800(2.5%)), and pure LiNbO_3_ calcined at 800 °C (LNbO-800). The marked reflections (23.7, 32.7 and 34.8 *2θ*) are attributed to coated LiNbO_3_.

**Figure 3:**
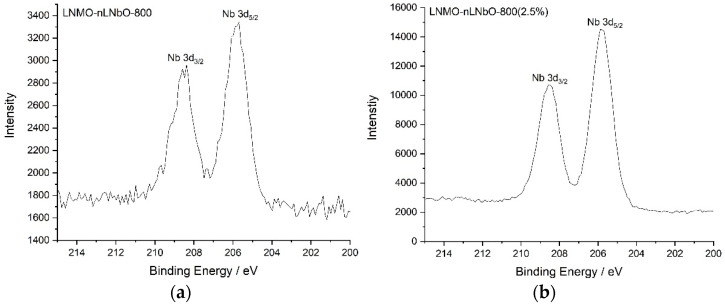
Nb 3d_3/2_ and Nb 3d_5/2_ XPS spectra of (**a**) LNMO-nLNbO-800 and (**b**) LNMO-nLNbO-800 (2.5%).

In order to better identify the morphology of the obtained crystalline particles and to verify the presence of the coating material on the surface, LNMO particles with increased LiNbO_3_ coating amount (~2.5%) were prepared and calcined at 800 °C. By comparing the SEM images of particles coated with different LiNbO_3_ quantity (Figure 1 and Figure S2), one can confidently admit that the coating material is present on the surface of the prepared LiMn_1.5_Ni_0.5_O_4_. The X-ray (Figure 2) and XPS (Figure 3) studies of the coated samples calcined at 800 °C also confirm the presence of LiNbO_3_ on the surface of LNMO particles. Taking into consideration the surface area of 0.3 m^2^g^−1^ for LNMO and theoretical density of LiNbO_3_ of 4.64 gcm^−3^, a layer thickness of ~3.6 nm for the 0.5 wt.% LiNbO_3_- and 18 nm for the 2.5 wt.% LiNbO_3_-coated LiMn_1.5_Ni_0.5_O_4_ particles could be assumed.

### 3.1. Scanning Electron Microscopy (SEM)

SEM images of the uncoated and coated LiMn_1.5_Ni_0.5_O_4_ at different temperatures are shown in Figure 1. The particles show a typical for the LNMO spinel regular truncated octahedral microstructure with large portions of (100) facets. Figure 1 shows the morphology of the pristine (Figure 1a), sol-gel 0.5 wt.% LiNbO_3_-coated LiMn_1.5_Ni_0.5_O_4_ particles sintered 400, 500, 600, and 800 °C (Figure 1b–e), and using the new method 0.5 wt.% LiNbO_3_-coated LiMn_1.5_Ni_0.5_O_4_ particles sintered at 800 °C (Figure 1f). In Figure 1a, smooth and clean surfaces are detected in the case of the pristine LNMO. After coating using sol-gel method, the surface of particles annealed at 400 and 500 °C gets coarser and fuzzier, showing that the LiMn_1.5_Ni_0.5_O_4_ particles are covered with a uniform LiNbO_3_ layer (see Figure 1b,c). From Figure 1d, at 600 °C, we can still observe numerous crystalline knolls on the surface of LiMn_1.5_Ni_0.5_O_4_ particles. At 800 °C (Figure 1e) some areas of the surface become slightly smoothly, but overall, it is still rude and bumpy. In contrast to the particles obtained by sol-gel method, the particles synthesized by the new method using hydrogen peroxide and calcined at 800 °C (Figure 1f) have a much smoother surface with well-defined edges and tips.

To find out which composition the obtained crystalline particles have on the surface, LNMO particles with increased LiNbO_3_ coating amount (2.5%) were prepared at 800 °C (Figure S2, right). Combined with the XRD (Figure 2) and XPS (Figure 3) results obtained for the sample coated with 2.5 wt.% LiNbO_3_, the surface morphology changes of the coated particles at different temperatures, using two different synthetic methods, confirm the formation of a coating layer of LiNbO_3_ on the surface of the LiMn_1.5_Ni_0.5_O_4_.

### 3.2. Powder X-ray Diffraction (XRD)

**Figure 4:**
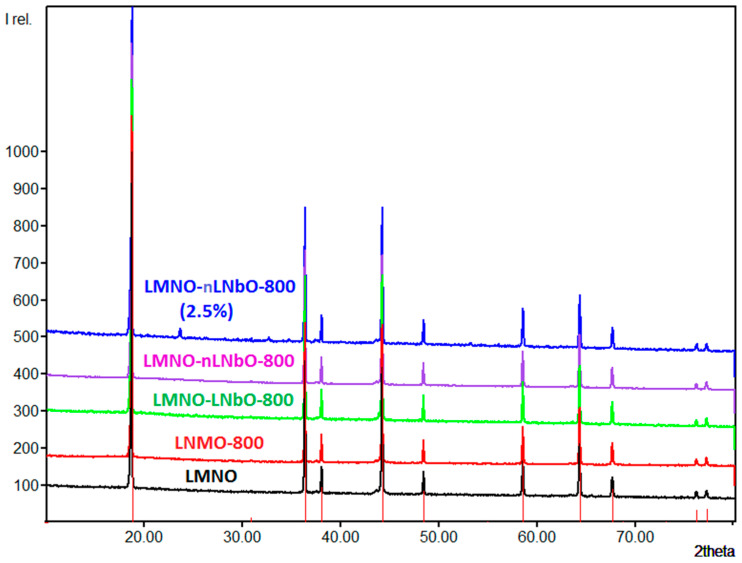
XRD patterns of uncoated LiMn_1.5_Ni_0.5_O_4_ (LNMO), uncoated and treated at 800 °C (LNMO-800), coated with 0.5 wt.% LiNbO_3_ using sol-gel method and calcined at 800 °C (LNMO-LNbO-800), coated with 0.5 wt.% LiNbO_3_ using new method and calcined at 800 °C (LNMO-nLNbO-800), and coated with 2.5 wt.% LiNbO_3_ using new method and calcined at 800 °C (LNMO-nLNbO-800(2.5%)).

The structures of uncoated and coated LiNi_0.5_Mn_1.5_O_4_, (with 0.5 and 2.5 wt.% LiNbO_3_) calcined at 800 °C were analyzed by XRD (Figure 4). The diffraction patterns can be indexed as mixed phases: in addition to the main LiNi_0.5_Mn_1.5_O_4_ cubic spinel, the obtained data reveal some minor impurity phase, which could be attributed to the well-known rock-salt Li*_x_*Ni*_1−x_*O [29,30]. The diffraction lines for LiNbO_3_ coating layer are not observed in the XRD pattern of 0.5 wt.% coated samples; due to low concentration of LiNbO_3_, its XRD reflections are shielded by the background of XRD patterns of bare LiNi_0.5_Mn_1.5_O_4_. Only when the coating reaction was done with 2.5 wt.% LiNbO_3_, from the XRD it is possible to observe and quantify the reflections of the coating material (Figure 4, top).

It is important to mention, that during the temperature treatment, especially at 800 °C, doping with niobium cations of the LNMO spinel could take place for the cations at the lithium and/or the Mn/Ni positions. Comparable substitutions have already been observed (e.g., for Ti, Al, Mg and Nb) and become evident on the basis of changed lattice parameter of LNMO [13,20,31,32,33,34,35]. Nb ions generally exhibit an oxidation state 5+ and have a similar ionic radius with Mn ions in the octahedral coordination environment. Their comparable sizes can facilitate ion substitution during calcination [36]. Such a substitution, which mostly takes place on the surface of the spinel, will not influence the oxidation state of Ni^2+^ ions, but will contribute to reduction in the oxidation state of Mn ions from 4+ to 3+ and appearance of a Mn^3+^-rich region. The XPS spectra of the 0.5 and 2.5 wt.%-coated LNMO samples, however, look essentially the same, indicating the presence of the same quantity of Mn^3+^ and Mn^4+^ in both compounds (Figure S3). Similarly, the initial charge/discharge profiles of the uncoated and LiNbO_3_-coated LNMO electrodes at 0.05 C (Figure S4) for the Mn^3+^/Mn^4+^ redox couple near 4.0 V exhibit no increased contribution from the Mn^3+^ ions.

From the XRD data of the lithium niobate coated compounds investigated herein, the doping with Nb^5+^ ions also does not seem to occur: the lattice parameters *a* of the LiNbO_3_-coated LNMO samples were refined by the Rietveld method, the results of which are listed in Table 1. Taking into account realistic estimated standard deviations according to Berar [37], no significant changes are observed, indicating that the cations of the coating material remain in the surface of the coating layer. For all samples, the rock salt secondary phase was refined to about 3 to 5 %. However, due to the overlap of the rock salt reflections with those of LNMO, a precise and reliable quantification can hardly be achieved. In contrast, for LNMO-nLNbO-800(2.5%) the phase fraction of LiNbO_3_ (space group *R*3*cH*, *a* = 514.8 pm, *c* = 1386.3 pm, [38]) could be refined easily and accurately to 2.0 %.

### 3.3. Electrochemistry

For the electrochemical studies, four samples were selected: the pristine, LNMO, the reference, LNMO-800 (pristine heated at 800 °C), LNMO-LNbO-800 and LNMO-nLNbO-800. The reason for this selection is based on our previous electrochemical temperature dependent studies, which show that LNMO improves its capacity retention and stability when it is treated at 800 °C [21]. Although LNMO treated at this temperature has a higher Mn^3+^ content, it shows a better cycling performance than the same samples treated at lower temperatures.

The electrochemical properties of uncoated and coated LNMO samples were evaluated using coin-type half-cells at voltage window of 3.5–5 V. The differential capacity versus potential (dQ/dV versus V) is shown in Figure S5 (supporting information). The broad and weak peak observed at ~4 V is associated with the Mn^3+^/Mn^4+^ couple, which indicate the presence in material of a disordered Fd-3m phase. Two well defined peaks in the voltage region of 4.5–4.8 V are related to the Ni^2+^/Ni^3+^ and Ni^3+^/Ni^4+^ redox processes.

In general, the uncoated active materials analyzed in this study show a relatively good long-term electrochemical performance. These properties appear to be dependent on the surface orientations of the octahedral spinel particles, which normally consist entirely of (111) surfaces. However, the materials examined here contain particles with large portions of truncated (100) surfaces, which stabilize the spinel structure and seem to promote better lithium-ion transport.

**Figure 5:**
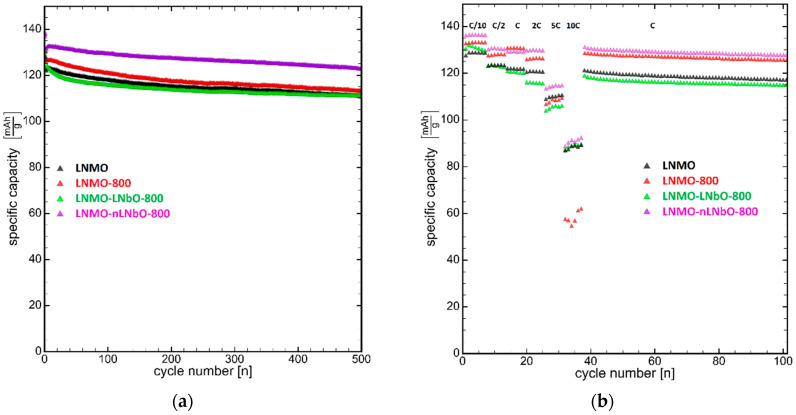
(**a**) Capacity retention test at charge-discharge rate at C/2-C (the first two cycles were done at 0.1 C) and (**b**) rate capability test (the first cycle was done at 0.05 C; charge rate was C/2 without holding the voltage and discharge rates were varied as indicated in the figure) of four samples: bare (LNMO), bare calcined at 800 °C (LNMO-800), coated using sol-gel method (LNMO-LNbO-800) and new method (LNMO-nLNbO-800) and calcined at 800 °C.

The long-term cycling performance of bare LNMO, LNMO-800, LNMO-LNbO-800 and LNMO-nLNbO-800 is given in Figure 5a. The cells were initially charged-discharged two cycles at 0.1 C, and then charged (at 0.5 C) and discharged (at 1 C) 500 times with the voltage of 3.5–5.0 V. The coulombic efficiency of all electrodes was ~99.8% at the charge-discharge cycle, which indicates a very high reversibility of the composite electrodes. The bare LNMO and LNMO-800 show initial capacities of ~122.0 and 126.0 mAhg^−1^, respectively. However, both electrodes show a comparable degree of capacity fading: after 500 cycles the remained capacity for the original LNMO is 91.1% and for the LNMO-800—90.0%. Although being coated and having a slightly smaller remained capacity of 89.5%, the obtained by sol-gel method LNMO-LNbO-800 has a similar initial capacity and behavior as the uncoated samples. On the other hand, the sample LNMO-nLNbO-800, obtained by the new method, shows not only a higher initial capacity (132.5 mAhg^−1^), but also a higher stability and capacity retention (93.5%).

The rate capability test of the pristine, thermally treated uncoated and coated spinels were run in the same range of 3.5–5.0 V and at different rates from 0.1 to 10 C, followed by a return to 1 C. (Figure 5b). The cells were first charged-discharged at a current density of 0.05 C for one cycle, six cycles at 0.1 C and six cycles at 0.5 C. Then they were charged with 0.5 C rate and then discharged at 1, 2, 5 and 10 C rates, respectively. After 10 C, the cells were further cycled at 1 C. Once the discharge rate increased from 0.1 to 10 C, the discharge capacity of the bare LNMO decreased from 128.9 to 88.6 mAhg^−1^, which was 68.7% capacity retention. The LNMO-800 shows 44.0% (from 133.2 to 58.6 mAhg^−1^), LNMO-LNbO-800—67.9% (from 131.1 to 89.1 mAhg^−1^) and LNMO-nLNbO-800 shows 66.8% (from 136.4 to 91.1 mAhg^−1^) capacity retentions. Hence, the high-rate capability of the LNMO cathodes was not improved by the coating and additional temperature treatment, and at high cycling rates they show almost the same capacity retention. However, the positive role of the coating is highlighted at lower cycling rates between 0.1 and 5 C, where the rate capability of the LNMO-nLNbO-800 composite material obtained by the new method is much better than in case of other three materials. Remarkably, once the LNMO-nLNbO-800 electrode was cycled at high rates and the current density proceeds back to 1 C, it recuperated its initial capacity. The recuperated capacity was even slightly bigger: 130.7 versus 129.2 mAhg^−1^. The recuperation of a bigger capacity indicates that the activation processes of the electrodes with LNMO coated by the new method take longer. The other three electrodes, LNMO, LNMO-800 and LNMO-LNbO-800, did not show such a result.

Since LNMO-LNbO-800 and LNMO-nLNbO-800 were thermally treated using the same temperature program, it is reasonable to expect the same crystallinity of the obtained coating material, and, respectively, the same electrochemical performance. This was not the case here. It is assumed that such a big difference in the initial capacity, capacity stability and rate capability could be due to the different morphology and homogeneity of the coating layer. Another reason could be a better contact between active material and coating layer in the sample obtained using our new method.

It must be emphasized that, using hydrogen peroxide as an activating agent, it is possible to generate a bigger number of oxygen ions per cation at the border between spinel and lithium niobate, and thus an increased degree of glass formation is achieved. In addition, the formation of highly covalent stable oxide frameworks at the interface through which the lithium can move freely cannot be ruled out. These two reasons can positively affect the ion conductivity at the crossing point and, respectively, the electrochemical performance.

In case of the sol-gel method, the lithium niobate coating layer also acts as a protection layer, preventing the active material from direct communication with electrolyte and suppressing the dissolution of metal ions. However, using this method, the surface activation is missing and the border between components is more like a mechanical mixture of two components and plays more of an obstacle role.

**Figure 6:**
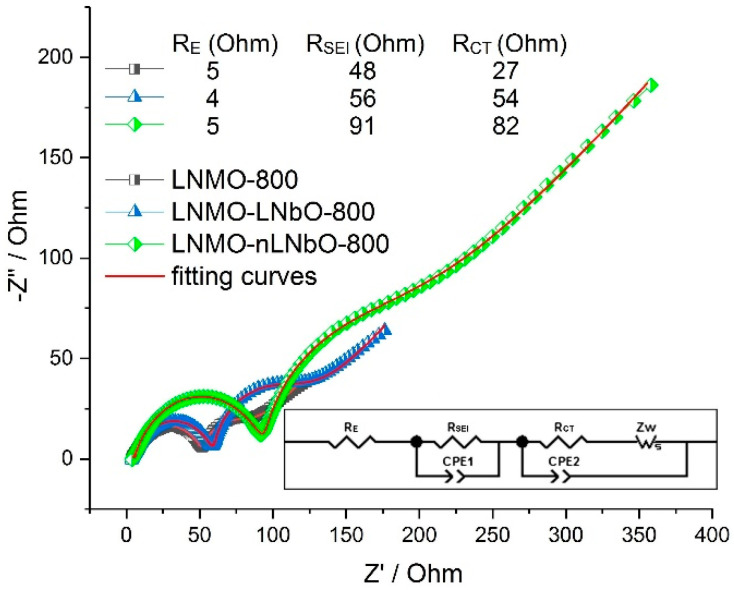
Electrochemical impedance spectra (EIS) plots after five cycles of pristine sample calcined at 800 °C (LNMO-800), coated using sol-gel method (LNMO-LNbO-800) and new method (LNMO-nLNbO-800) and calcined at 800 °C. Inset: the equivalent circuit for EIS results fitting.

Thus, from the results presented above it can be clearly seen that the lithium niobate oxide coating demonstrates a new method for enhancing the high voltage cathode performance and shows promising results. Hence, an enhanced conductivity and a reduced inner resistance are expected in the materials studied in this work. Usually this is the case: a huge number of reported coated active materials show these properties [39,40]. To confirm our expectation, electrochemical impedance spectra (EIS) were collected with an AC voltage of 5 mV amplitude in the frequency range of 500 kHz to 10 mHz. A Nyquist plot is normally separated by an electrolyte resistance (R_E_), two semi-circles of solid electrolyte interface (SEI) resistance (R_SEI_) and charge transfer resistance (R_CT_), and a mass transfer (Li^+^) slope of Warburg resistance (Z_W_). Figure 6 shows the AC impedance test results for LNMO-800, LNMO-LNbO-800 and LNMO-nLNbO-800 at the 5th cycle (0.5 C, SOC 50% (4.25 V)), respectively. The fitted results are drawn as solid lines, and the resistance values calculated from the fitting results are presented as inset. Generally, the SEI layer is expected to form in the first five charge-discharge cycles of LNMO electrodes. It can be undoubtedly seen that the impedance of LiNbO_3_-coated samples is bigger than that of the uncoated sample. This result is in contradiction with the results reported in the literature [41,42], that show that a coating material which improves the battery’s performance will reduce its impedance. Actually, the result obtained in our study is reasonable to expect: the resistance of a 10–20 nm thick LiNbO_3_ oxide solid layer will be much bigger than the resistance of a SEI layer, which, after five cycles, will be poorly developed and the charge transfer kinetic will not be considerably weakened. The real cause of such a contradiction is not currently clear and will be analyzed in other studies.

We would also like to mention that there is a big number of reports showing diverse coated active materials with improved performance due to different phosphate or oxide coatings [39,40,41,42]. To distinguish between the contributions from the coating process itself and the effect of composite present in the coating system, it is advisable to produce a process reference compound. Such a material/uncoated sample (in our case LNMO-800), treated at the same conditions as the coated one, is useful for the reflection of the process influences. Unfortunately, such reference compounds are rarely used [43,44] in the published studies and the published results are not very suitable for us to define the real reason of the discrepancies between our own and published impedance data.

## 4. Conclusions

LiNbO_3_ coating layers on LNMO spinel cathode were successfully introduced into lithium-ion batteries using a sol-gel and new chemically activated coating reactions. The LiNbO_3_-coated LiNi_0.5_Mn_1.5_O_4_ material by the new method, using hydrogen peroxide as activating reagent, exhibited higher capacity, cycling stability and higher rate capability than the pristine and reference samples.

The process of deposition of the LiNbO_3_ coating was tracked and documented by SEM, XRD and XPS studies. The obtained results support the successful deposition of the LiNbO_3_ coating; particularly, the temperature dependent crystallization process of the coating materials monitored by SEM clearly indicates the presence of the coating sample on the LNMO surface.

Using our new method, it is possible to circumvent the poor contact and large charge transfer resistance between the coating and active material. It looks like the surface activation and simultaneous coating results in an enhanced homogeneity, much better contact and communication between the materials, positively affecting the ion conductivity and electrochemical performance. The successful combination of an ion conducting coating layer with a high-voltage cathode material makes the proposed synthetic method very promising for the protection of cathode materials and provides a clear direction for designing next-generation large-scale lithium-ion batteries.

It is known that using atomic layered deposition (ALD) techniques it is possible to produce uniform coating layers with appropriate coating thickness. The proposed coating method combines the ALD coating quality with a low-cost synthetic procedure. In addition, the offered method is easy to be scaled up to industrial grade. Such a combination plays a significant role for manufacturers.

The proposed method can also be extended on other ion conducting solid electrolytes and cathode materials. Such work is under way and will be reported in the near future.

## Figures and Tables

**Table 1:** Results of the Rietveld refinement of the lattice parameter *a* of LNMO and phase fraction of LiNbO_3_ after coating with 2.5 wt.% LiNbO_3_.

Sample	Lattice Parameter, *a* (pm)	*x* (LiNbO_3_)
LNMO	818.10(8)	-
LNMO-800	817.93(7)	-
LNMO-LNbO-800	817.95(8)	-
LNMO-nLNbO-800	818.06(7)	-
LNMO-nLNbO-800 (2.5%)	817.77(12)	2.00%

## Data Availability

The data presented in this study are available on request from the corresponding author.

## Supplementary Materials

The following are available online at https://www.mdpi.com/2079-4991/11/2/548/s1, Figure S1: XRD patterns of LiNbO_3_ calcined at 800 °C (LNbO-800), Figure S2: SEM diagrams of samples LMNO-nLNbO-800 with 0.5% LiNbO_3_ (left) and LMNO-nLNbO-800(2.5%) with 2.5% LiNbO_3_ (right), Figure S3: Mn 2p_1/2_ and Mn 2p_3/2_ XPS spectra of (left) LNMO-nLNbO-800 and (right) LNMO-nLNbO-800 (2.5%), Figure S4: Initial charge-discharge voltage profiles at 0.05 C of uncoated and LiNbO_3_-coated LNMO samples in the potential range of 3.5–5.0 V, Figure S5: (Left) Differential capacity versus potential (dQ/dV versus V) between 3.5 and 5 V (100th cycle). (Right) The zoomed 4.54–4.83 V region, Figure S6: SEM diagrams with EDS results of sample LNMO-nLNbO-800 (2.5%).

## References

[1] Alcantra R., Jaraba M., Lavela P., Tirado J.L. (2004). X-ray diffraction and electrochemical impedance spectroscopy study of zinc coated LiNi_0.5_Mn_1.5_O_4_ electrodes. J. Electroanal. Chem..

[2] Aurbach D., Markovsky B., Talyossef Y., Salitra G., Kim H.-J., Choi S. (2006). Studies of cycling behavior, ageing, and interfacial reactions of LiNi_0.5_Mn_1.5_O_4_ and carbon electrodes for lithium-ion 5-V cells. J. Power Sources.

[3] Lazarraga M.G., Pascual L., Gadjov H., Kovacheva D., Petrov K., Amarilla J.M., Rojas R.M., Martin-Luengo M.A., Rojo J.M. (2004). Nanosize LiNi_y_Mn_2−y_O_4_ (0 < y ≤ 0.5) spinels synthesized by a sucrose-aided combustion method. Characterization and electrochemical performance. J. Mater. Chem..

[4] Arrebola J.C., Caballero A., Cruz M., Hernan L., Morales J., Castellon E.R. (2006). Crystallinity Control of a Nanostructured LiNi_0.5_Mn_1.5_O_4_ Spinel via Polymer-Assisted Synthesis: A Method for Improving Its Rate Capability and Performance in 5 V Lithium Batteries. Adv. Funct. Mater..

[5] Goodenough J.B., Kim Y. (2011). Challenges for rechargeable batteries. J. Power Sources.

[6] Wang H.L., Tan T.A., Yang P., Lai M.O., Lu L. (2011). High-Rate Performances of the Ru-Doped Spinel LiNi_0.5_Mn_1.5_O_4_: Effects of Doping and Particle Size. J. Phys. Chem. C.

[7] Belharouak I., Lu W., Vissers D., Amine K. (2006). Safety characteristics of Li(Ni_0.8_Co_0.15_Al_0.05_)O_2_ and Li(Ni_1/3_Co_1/3_Mn_1/3_)O_2_. Electrochem. Commun..

[8] Dahn J.R., Fuller E., Obrovac M., Vonsacken U. (1994). Thermal stability of Li_x_CoO_2_, Li_x_NiO_2_ and λ-MnO_2_ and consequences for the safety of Li-ion cells. Solid State Ionics.

[9] Sun Y.K., Hong K.J., Prakash J., Amine K. (2002). Electrochemical performance of nano-sized ZnO-coated LiNi_0.5_Mn_1.5_O_4_ spinel as 5 V materials at elevated temperatures. Electrochem. Commun..

[10] Talyosef Y., Markovsky B., Salitra G., Aurbach D., Kim H.J., Choi S. (2005). The study of LiNi_0.5_Mn_1.5_O_4_ 5 V cathodes for Li-ion batteries. J. Power Sources.

[11] Markovsky B., Talyosef Y., Salitra G., Aurbach D., Kim H.J., Choi S. (2004). Cycling and storage performance at elevated temperatures of LiNi_0.5_Mn_1.5_O_4_ positive electrodes for advanced 5 V Li-ion batteries. Electrochem. Commun..

[12] Kong J.-Z., Ren C., Tai G.-A., Zhang X., Li A.-D., Wu D., Li H., Zhou F. (2014). Ultrathin ZnO coating for improved electrochemical performance of LiNi_0.5_Co_0.2_Mn_0.3_O_2_ cathode material. J. Power Sources.

[13] Shim J., Lee S., Park S. (2014). Effects of MgO Coating on the Structural and Electrochemical Characteristics of LiCoO_2_ as Cathode Materials for Lithium Ion Battery. Chem. Mater..

[14] Kim M.G., Cho J. (2008). Air stable Al_2_O_3_-coated Li_2_NiO_2_ cathode additive as a surplus currentconsumer in a Li-ion cell. J. Mater. Chem..

[15] Wang J.-H., Wang Y., Guo Y.-Z., Ren Z.-Y., Liu C.-W. (2013). Effect of heat-treatment on the surface structure and electrochemical behavior of AlPO_4_-coated LiNi_1/3_Co_1/3_Mn_1/3_O_2_ cathode materials. J. Mater. Chem. A.

[16] Huang B., Li X., Wang Z., Guo H., Shen L., Wang J. (2014). A comprehensive study on electrochemical performance of Mn-surface-modified LiNi_0.8_Co_0.15_Al_0.05_O_2_ synthesized by an in situ oxidizing-coating method. J. Power Sources.

[17] Glass A., Nassau K., Negran T. (1978). Ionic conductivity of quenched alkali niobate and tantalate glasses. J. Appl. Phys..

[18] Zhang Z.-J., Chou S.-L., Gu Q.-F., Liu H.-K., Li H.-J., Ozawa K., Wang J.-Z. (2014). Enhancing the high rate capability and cycling stability of LiMn_2_O_4_ by coating of solid-state electrolyte LiNbO_3_. ACS Appl. Mater. Interfaces.

[19] Sun W., Xie M., Shi X., Zhang L. (2015). Study of new phases grown on LiNbO_3_ coated LiCoO_2_ cathode material with an enhanced electrochemical performance. Mater. Res. Bull..

[20] Kim H., Byun D., Chang W., Jung H.-G., Choi W. (2017). A nano-LiNbO_3_ coating layer and diffusion-induced surface control towards high-performance 5 V spinel cathodes for rechargeable batteries. J. Mater. Chem. A.

[21] Mereacre V., Bohn N., Müller M., Indris S., Bergfeldt T., Binder J.R. (2021). Improved performance of high-voltage Li-ion batteries using a novel chemically activated coating process. Mater. Res. Bull..

[22] Hua W., Wu Z., Chen M., Knapp M., Guo X., Indris S., Binder J.R., Bramnik N.N., Zhong B., Guo H. (2017). Shape-controlled synthesis of hierarchically layered lithium transition-metal oxide cathode materials by shear exfoliation in continuous stirred-tank reactors. J. Mater. Chem. A.

[23] Antoniassi B., González A.H.M., Fernandes S.L., Graeff C.F.O. (2011). Microstructural and electrochemical study of La_0.5_Li_0.5_TiO_3_. Mater. Chem. Phys..

[24] Bohnke C., Regrag B., Le Berre F., Fourquet J.-L., Randrianantoandro N. (2005). Comparison of pH sensitivity of lithium lanthanum titanate obtained by sol-gel synthesis and solid state chemistry. Solid State Ionics.

[25] Kobylyanskaya S., Gavrilenko O., Belous A. (2013). Synthesis of nanosized (Li,La){Ti,Nb,Ta}O_3_ particles using the sol-gel method. Russ. J. Inorg. Chem..

[26] Popovici I.C., Chirila E., Popescu V., Ciupina V., Prodan G. (2007). Sol-gel preparation and characterization of perovskite lanthanum lithium titanate. J. Mater. Sci..

[27] Zhu Y.-R., Yuan J., Zhu M., Hao G., Yi T.-F., Xie Y. (2015). Improved electrochemical properties of Li_4_Ti_5_O_12_–Li_0.33_La_0.56_TiO_3_ composite anodes prepared by a solid-state synthesis. J. Alloys Compd..

[28] Zhang H., Yang T., Han H., Song D., Shi X., Zhang L., Bie L. (2017). Enhanced electrochemical performance of Li_1.2_Ni_0.13_Co_0.13_Mn_0.54_O_2_ by surface modification with the fast lithium-ion conductor Li-La-Ti-O. J. Power Sources.

[29] Zhong Q., Bonakdarpour A., Zhang M., Gao Y., Dahn J.R. (1997). Synthesis and Electrochemistry of LiNi_x_Mn_2-x_O_4_. J. Electrochem. Soc..

[30] Alca’ntara R., Jaraba M., Lavela P., Tirado J.L. (2002). Optimizing preparation conditions for 5 V electrode performance, and structural changes in Li_1−_*_x_*Ni_0.5_Mn_1.5_O_4_ spinel. Electrochim. Acta.

[31] Schroeder M., Glatthaar S., Geßwein H., Winkler V., Bruns M., Scherer T., Chakravadhanula V.S.K., Binder J.R. (2013). Post-doping via spray-drying: A novel sol-gel process for the batch synthesis of doped LiNi_0.5_Mn_1.5_O_4_ spinel material. J. Mater. Sci..

[32] Wang H., Ben L., Yu H., Chen Y., Yang X., Huang X. (2017). Understanding the effects of surface reconstruction on the electrochemical cycling performance of the spinel LiNi_0.5_Mn_1.5_O_4_ cathode material at elevated temperatures. J. Mater. Chem. A.

[33] Liu M.-H., Huang H.-T., Lin C.-M., Chen J.-M., Liao S.-C. (2014). Mg gradient-doped LiNi_0.5_Mn_1.5_O_4_ as the cathode material for Li-ion batteries. Electrochim. Acta.

[34] Sun P., Ma Y., Zhai T., Li H. (2016). High performance LiNi_0.5_Mn_1.5_O_4_ cathode by Al-coating and Al^3+^-doping through a physical vapor deposition method. Electrochim. Acta.

[35] Cho J., Kim Y.J., Park B. (2000). Novel LiCoO_2_ cathode material with Al_2_O_3_ coating for a Li ion cell. Chem. Mater..

[36] Li J., Tian Y., Xu C. (2012). Influence of Nb^5+^ Doping on Structure and Electrochemical Properties of Spinel Li_1.02_Mn_2_O_4_. J. Mater. Sci. Technol..

[37] Berar J.F., Lelann P.E. (1991). E.S.D.’s and estimated probable error obtained in Rietveld refinements with local correlations. J. Appl. Cryst..

[38] Abrahams S.C., Reddy J.M., Bernstein J.L. (1966). Ferroelectric lithium niobate. 3. Single crystal x-ray diffraction study at 24 °C. J. Phys. Chem. Solids.

[39] Okada K., Machida N., Naito M., Shigematsu T., Ito S., Fujiki S., Nakano M., Aihara Y. (2014). Preparation and electrochemical properties of LiAlO_2_-coated Li(Ni_1/3_Mn_1/3_Co_1/3_)O_2_ for all-solid-state batteries. Solid State Ionics.

[40] Zhang L.-L., Wang J.-Q., Yang X.-L., Liang G., Li T., Yu P.-L., Ma D. (2018). Enhanced Electrochemical Performance of Fast Ionic Conductor LiTi_2_(PO_4_)_3_-Coated LiNi_1/3_Co_1/3_Mn_1/3_O_2_ Cathode Material. ACS Appl. Mater. Interfaces.

[41] Li L., Chen Z., Zhang Q., Xu M., Zhou X., Zhua H., Zhang K. (2015). A hydrolysis-hydrothermal route for the synthesis of ultrathin LiAlO_2_-inlaid LiNi_0.5_Co_0.2_Mn_0.3_O_2_ as a high-performance cathode material for lithium ion batteries. J. Mater. Chem. A.

[42] Wen W., Yang X., Wang X., Ge L., Shu H. (2015). Improved electrochemical performance of the spherical LiNi_0.5_Mn_1.5_O_4_ particles modified by nano-Y_2_O_3_ coating. J. Solid State Electrochem..

[43] Dannehl N., Steinmüller S.O., Szabó D.V., Pein M., Sigel F., Esmezjan L., Hasenkox U., Schwarz B., Indris S., Ehrenberg H. (2018). High-Resolution Surface Analysis on Aluminum Oxide-Coated Li_1.2_Mn_0.55_Ni_0.15_Co_0.1_O_2_ with Improved Capacity Retention. ACS Appl. Mater. Interfaces.

[44] Lee Y., Lee J., Lee K.Y., Mun J., Lee J.K., Choi W. (2016). Facile formation of a Li_3_PO_4_ coating layer during the synthesis of a lithium-rich layered oxide for high-capacity lithium-ion batteries. J. Power Sources.

