# Supplementary material for: Enhancing the Stability of LiNi0.5Mn1.5O4 by Coating with LiNbO3 Solid-State Electrolyte: Novel Chemically Activated Coating Process versus Sol-Gel Method
Source: Nanomaterials (Basel). 2021 Feb 22;11(2):548. doi: 10.3390/nano11020548 (PMC7926566; doi:10.3390/nano11020548)
Supplement: Supplementary file 1 [file nanomaterials-11-00548-s001.pdf]

Supplementary Materials:

Enhancing the Stability of $LiNi_{0.5}Mn_{1.5}O_4$ by Coating with $LiNbO_3$ Solid-State Electrolyte: Novel Chemically Activated Coating Process Versus Sol-Gel Method

Valeriu Mereacre [1,*], Pirmin Stüble [1,2], Ahmad Ghamlouche [1] and Joachim R. Binder [1]

[1] Institute for Applied Materials, Energy Storage Systems, Karlsruhe Institute of Technology, Hermann-von-Helmholtz-Platz 1, D-76344, Eggenstein-Leopoldshafen, Germany; pirmin.stueble@kit.edu (P.S.); ahmad.ghamlouche@kit.edu (A.G.); joachim.binder@kit.edu (J.R.B.)

[2] Helmholtz Institute Ulm, D-89081, Ulm, Germany

* Correspondence: valeriu.mereacre@kit.edu

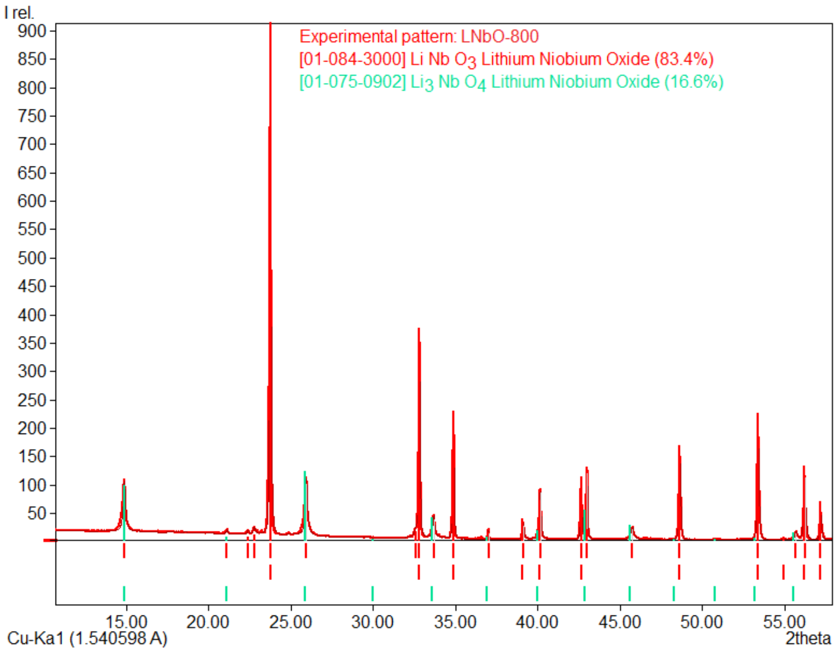

Figure S1. XRD patterns of $LiNbO_3$ calcined at 800°C (LNbO-800).

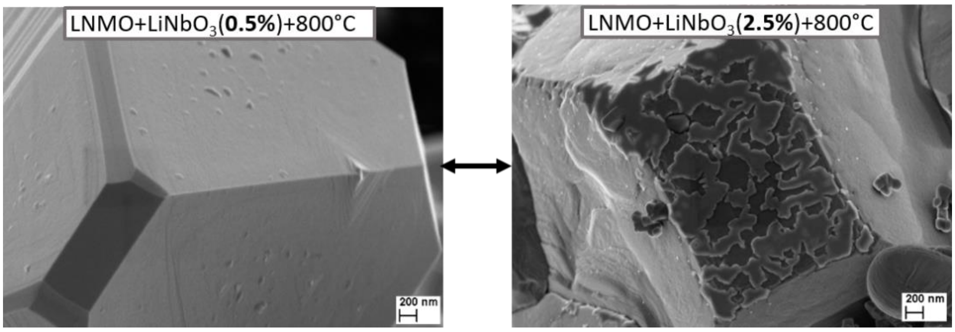

Figure S2. SEM diagrams of samples LMNO-nLNbO-800 with 0.5% $LiNbO_3$ (left) and LMNO-nLNbO-800(2.5%) with 2.5% $LiNbO_3$ (right).

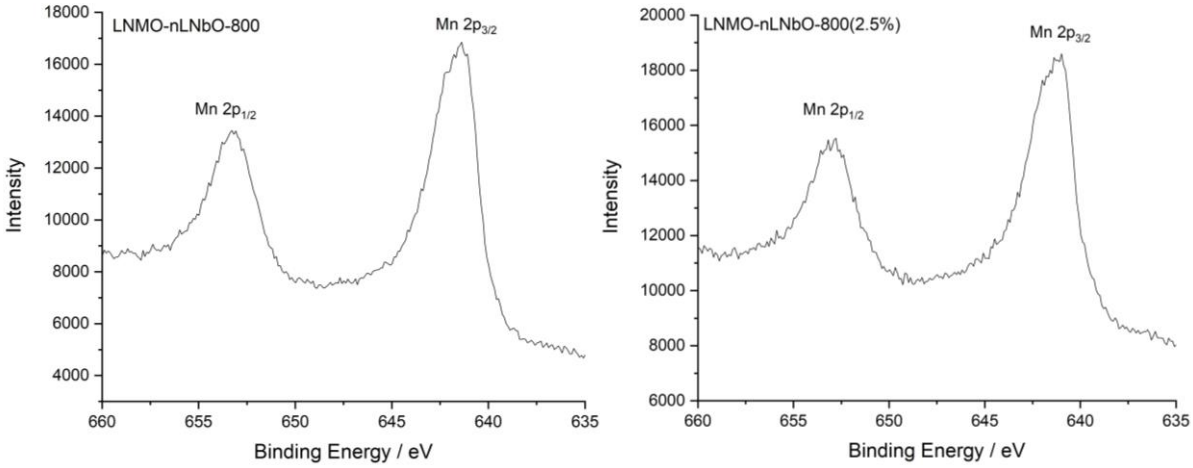

Figure S3. Mn $2p_{1/2}$ and Mn $2p_{3/2}$ XPS spectra of (left) LNMO-nLNbO-800 and (right) LNMO-nLNbO-800(2.5%)).

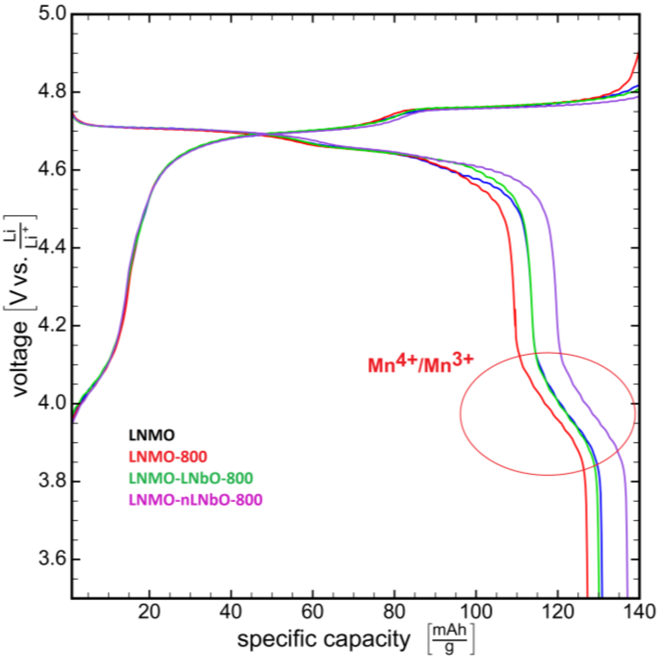

Figure S4. Initial charge-discharge voltage profiles at 0.05 C of uncoated and LiNbO3-coated LNMO samples in the potential range of 3.5–5.0 V.

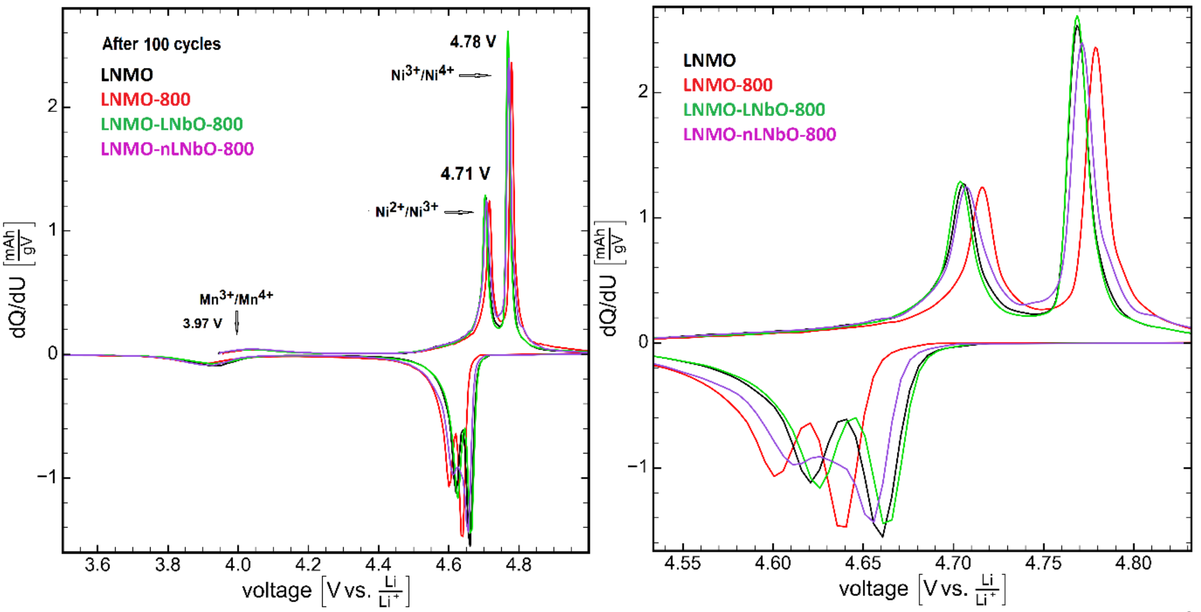

Figure S5 (Left) Differential capacity versus potential (dQ/dV versus V) between 3.5 and 5 V (100th cycle). (Right) The zoomed 4.54 – 4.83 V region.

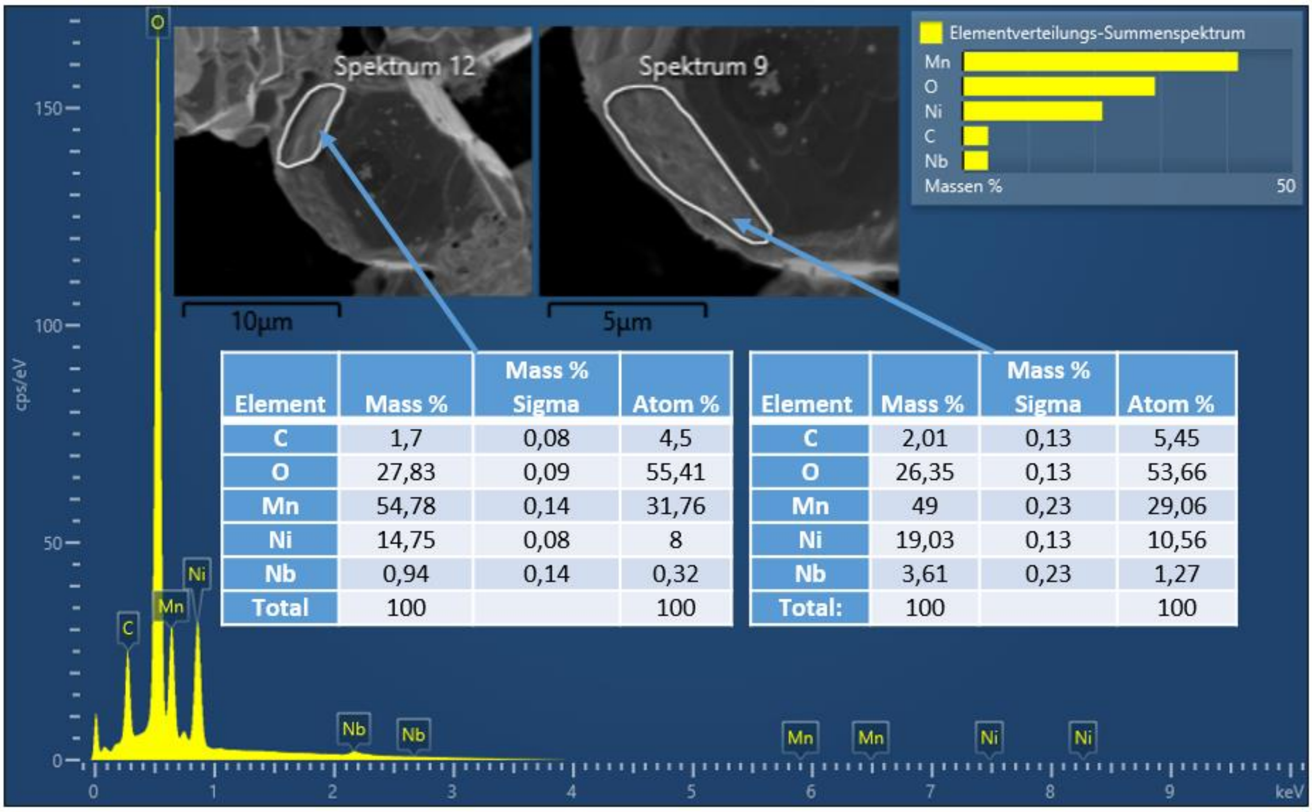

Element	Mass %	Mass % Sigma	Atom %
C	1,7	0,08	4,5
O	27,83	0,09	55,41
Mn	54,78	0,14	31,76
Ni	14,75	0,08	8
Nb	0,94	0,14	0,32
Total	100		100

Element	Mass %	Mass % Sigma	Atom %
C	2,01	0,13	5,45
O	26,35	0,13	53,66
Mn	49	0,23	29,06
Ni	19,03	0,13	10,56
Nb	3,61	0,23	1,27
Total:	100		100

Figure S6. SEM diagrams with EDS results of sample LNMO-nLNbO-800(2.5%).
